# Some Practical Points in Porcelain Work

**Published:** 1896-05

**Authors:** A. C. Mc Alpin

**Affiliations:** Warren, Pa.


ARTICLE XI.

### SOME PRACTICAL POINTS IN PORCELAIN WORK.

BY DR. A. C. MC ALPIN, WARREN, PA.

Read at the Twenty-seventh Union Dental Convention of the Sixth, Seventh and Eighth District Dental Societies of the State of New York, held at Binghamtown, Oct. 29, 30 and 31, 1895.

It is not the purpose of this paper to give, in elaborate detail, a description of all the operations possible in what is specified as porcelain work, but simply briefly to review some of those practical operations which have come within my experience during the past five years. Porcelain work well done, is very durable and artistic in appearance; done badly, it is more apparently discreditable to the operator than any other class of work in operative dentistry.

A conspicuous and radical fault found in porcelain operations is the carelessness with which colors are selected, the universal tendency being to choose too light and too yellow shades. The operator, disregarding the habits of his patient with regard to his teeth, selects porcelain facings and fillings to match the newly-polished, or dry teeth, when oftentimes he well knows that the patient will allow the teeth to lapse into the old condition, and that the porcelain will not gather a like deposit with the natural teeth. A highly glazed porcelain facing on a crown should rarely be left. It may be remedied by deadening the surface with sandpaper disks, and polishing with buff and pumice. In porcelain fillings the color of the cement used as the retaining medium influences the shade, and care must be taken in its selection.

The experienced operator in porcelain work will abandon small approximal fillings, and confine himself to labial fillings, conspicuous corners, and tips on teeth eroded, or worn to cup shape. A porcelain filling involves the preparation of the cavity in such a way that the matrix can be easily withdrawn, without affecting its shape, and the completed filling can be inserted. Very acute marginal angles in the fillings should be avoided. The use of separators enters largely in the succeesful insertion of approximal fillings, and simplifies an operation when many times it would seem impossible to insert the filling with a sufficient body to it for anchorage.

To form a porcelain filling, take platinum plate of a guage as thin as it is possible to use without tearing, anneal it to white heat, cool and place it over the cavity, forming a matrix by pressing it to the walls with cotton pellets, rolled hard, rubber points and burnishers, and getting the matrix well defined by burnishing it to the marginal lines and over the surface of the tooth around the cavity, the latter to stiffen it and facilitate handling. Then fill the matrix with porcelain body to match the natural tooth, and fuse. Remove the platinum, make slight retaining grooves in the body of the filling with a diamond disk, these grooves to abut as nearly as possible upon similar ones made in the side walls of the cavity. A mere scratch in labial cavities will suffice. Then dry the cavity and filling, and insert with a thin mixture of cement, cover with sandarac, and and allow to set well before removing the surplus cement. Extreme care should be taken that the marginal lines should be so perfect that subsequent grinding on them will be unnecessary. In filling cervico-labial cavities, pink porcelain body can be used to take the place of gum tissue, lost through recession. In large corners and tips, oftentimes short double-headed pins should be made and used, one head in the filling, the other as an anchorage button in the cavity.

The operations next described are those in which porcelain work is most valuable. In these the jacket crown is used. Teeth that have been repeatedly filled with metals and have as often failed, can be restored to their natural appearance and use without disturbing the living pulp. When the natural crown has decayed beyond the metal filling, and the tooth is yet alive, or the crown is undeveloped, or is irregular in form, or twisted in its socket, or slightly out of position, or worn away by friction or erosion, it can be crowned and regulated without sacrificing the pulp, and with a crown whose facing has the firmest attachments throughout its entire palatine surface, thus making the strongest porcelain-faced crown known. These crowns are so constructed that easy access to the pulp in direct line with the root canal can be had in case of subsequent trouble. They are commonly used only on the twenty anterior teeth, as gold crowns are usually practicable for the molars. The crown is made as follows:

Reduce the natural crown to a size which will admit of a twenty-eight American guage platinum plate over the entire palatine and approximal surfaces, and a twenty two gauge porcelain facing. Obliterate the cervical ridge, then fit the cervical circumference of the stub with a thirty gauge platinum cylindrical tube (in making which, lap the metal ends a little), solder this with pure gold, using as little as possible, place the tube on the stub, mark it on a line with the palatine surfaces of the approximating teeth and gums, and grind the tube inside the lines with a dry stone, to such thinness that it can be burnished to the stub without affecting the cervical adaptation. Then solder to this surface, with pure gold (using as little as possible), a twenty-eight American gauge platinum plate, *this* to form the entire palatine and about half the approximal surfaces of the crown. Then mark and grind the labial surface of the tube and adapt to the stub by pressure, without burnishing. use for this an old amalgam serrated plugger, that the ground surface may remain rough for the adhesion of the porcelain, then grind the back of the veneer facing so that when placed on the shell, it will be in the position required when finished. All platinum surfaces to which porcelain body is to adhere, should be roughened with a dry stone. Remove the shell from the stub, heat it to white heat, to cleanse and prevent carbonation, which might discolor the facing, and place the facing on the shell in correct position, with the porcelain body between, and dry well before baking. Bake to a slight gloss only on the porcelain body, cool slowly and finish the same as in Richmond crown work.

In attaching a bridge tooth to a jacket crown, allow the heavy palatine backing to extend across the space to be filled, roughen its labial surface with a dry stone, curl the edges slightly to stiffen them, and proceed with a facing as in the jacket crown.

For longer bridges, the jacket minus the porcelain can be made, but with pins attached to the labial surface, the bridge teeth soldered to them as in ordinary bridge-work. The facings can be put on the shells by the method explained, in the attaching of a cup facing. For replacing a lost facing from a bridge, which is broken, burnish a thirty-two gauge platinum plate to the bridge backing, with holes to allow pins to go through the plate, solder to this with pure gold, (using as little gold as possible) two tubes of platinum made to fit closely over the pins. Attach to this the old facing or a duplicate, in the same manner as in attaching the facing to the jacket crown. Cement this on and you have a permanent repair without the trouble of taking off the bridge.

To those aggressive who, with impunity, knock out the nerve and never let lose from subsequent trouble more than the old original two per cent., this jacket crowning will seem superfluous, but to those "meek and lowlies," who advocate the preservation of the pulp whenever possible, and wish to be free from the two per cent. mortality, and at the same time wish to feel the satisfaction of doing better work, this method of crowning will commend itself.

Some other practical uses for porcelain work may be found in fusing pins or porcelain buttons on blocks, repairing cracked blocks, contouring teeth and portions of gum sections, making two blocks continuous, coloring teeth with mineral stains or bodies, to match anomalous shades, painting gold fillings on artificial crowns, etc. In post crowns, perfect adaptation to the root with porcelain can be had by fastening the screw post in the root first, and proceeding as in the attachments of a cup facing, making the entire crown with the exception of the veneer facing, of the porcelain body.

Porcelain work enters largely as an aid in orthodontia, and a little experience in the jacket and bridge work will enable the operator to discern those cases in which it can be used.

I trust that the discussion of this paper will clear any ambiguous points and bring out those necessarily omitted by its brevity.

